# Energy Transfer between Tm-Doped Upconverting Nanoparticles and a Small Organic Dye with Large Stokes Shift

**DOI:** 10.3390/bios9010009

**Published:** 2019-01-08

**Authors:** Anna López de Guereñu, Philipp Bastian, Pablo Wessig, Leonard John, Michael U. Kumke

**Affiliations:** 1 Department of Chemistry (Physical Chemistry), University of Potsdam, Karl-Liebknecht-Str. 24-25, 14476 Potsdam, Germany; alopes1@mail.ru (A.L.d.G.); pbastian@uni-potsdam.de (P.B.); 2 Department of Chemistry (Organic Chemistry), University of Potsdam, Karl-Liebknecht-Str. 24-25, 14476 Potsdam, Germany; wessig@uni-potsdam.de (P.W.); lejohn@uni-potsdam.de (L.J.)

**Keywords:** resonance energy transfer, DBD dye, core shell UCNP, time-resolved luminescence

## Abstract

Lanthanide-doped upconverting nanoparticles (UCNP) are being extensively studied for bioapplications due to their unique photoluminescence properties and low toxicity. Interest in RET applications involving UCNP is also increasing, but due to factors such as large sizes, ion emission distributions within the particles, and complicated energy transfer processes within the UCNP, there are still many questions to be answered. In this study, four types of core and core-shell NaYF_4_-based UCNP co-doped with Yb^3+^ and Tm^3+^ as sensitizer and activator, respectively, were investigated as donors for the Methyl 5-(8-decanoylbenzo[1,2-d:4,5-d′]bis([1,3]dioxole)-4-yl)-5-oxopentanoate (DBD-6) dye. The possibility of resonance energy transfer (RET) between UCNP and the DBD-6 attached to their surface was demonstrated based on the comparison of luminescence intensities, band ratios, and decay kinetics. The architecture of UCNP influenced both the luminescence properties and the energy transfer to the dye: UCNP with an inert shell were the brightest, but their RET efficiency was the lowest (17%). Nanoparticles with Tm^3+^ only in the shell have revealed the highest RET efficiencies (up to 51%) despite the compromised luminescence due to surface quenching.

## 1. Introduction

Resonance energy transfer (RET) based on Coulombic interaction is nowadays a very frequently used fundamental photophysical principle to determine the distance between molecules on a nanometer scale [1,2]. It is applied to monitor conformational changes in (bio)molecules or to quantitatively determine a binding interaction between two molecules (e.g., antigen–antibody), both are connected to a change in relevant distance [3]. This distance alteration can be monitored with high sensitivity (even on a single molecule level) in real-time, which makes its application even more valuable. In order to create a RET-based sensing scheme, the introduction of suitable chromophores with matched photophysical properties is mandatory. Extrinsic as well as intrinsic chromophores are used, the latter especially in biological systems in which extrinsic labelling of molecules of interest is sometimes difficult without introducing larger disturbances to the system under investigation. In the RET process, energy transfer from one chromophore (donor) to the other (acceptor) is radiationless. In order to be effective, the spectral match (expressed as the spectral overlap integral between donor emission and acceptor absorption), the orientation of the transition dipole moments between donor and acceptor (orientation factor k^2^), and the emission quantum yield of the donor must be large [4]. Depending on the specific parameter, distances accessible in RET are in the range of 1–10 nm [5].

In the last decades luminescent nanoparticles have emerged as powerful competitors to organic chromophores, due to their photobleaching, ns-fluorescence lifetimes, and chemical instability of organic chromophores [6]. For instance, semiconductor quantum dots (Qdots) are claimed to have the following advantages in the context of biosensing: superior photostability, high brightness, and sharp emission lines, of which the latter can be tailored by adjusting the size of the Qdots (size quantization effect). Qdots have been successfully integrated in RET-based sensing schemes serving as donor or acceptor. Their sharp, well-defined emission lines have made it possible to establish multiparameter detection schemes [7]. Despite the advantages of Qdots, they have some drawbacks, which have limited their broader application—for instance, toxicity, instability (dissolution), and so-called “blinking” (random fluctuations in Qdot photoluminescence) [8,9].

Graphene quantum dots (GQdots), or graphene nanosheets with a diameter under 20 nm, are characterized by tunable optical properties, high brightness, good photostability, and long fluorescence lifetimes, which makes them promising RET donors. They are also more biocompatible than Qdots, making them attractive for biomedical applications. However, real-world application of GQdots for (bio)sensing is restricted by short luminescence lifetimes, excitation-dependent effects of photoluminescence and a lack of control over their dimensions and surface chemistry [6].

Lanthanide-doped upconverting nanoparticles (UCNP) are non-toxic, non-blinking, and chemically inert. Their advantages also include low background fluorescence, large anti-Stokes shifts, long (up to hundreds of µs) luminescence lifetimes as well as outstanding photostability. These properties make UCNP promising for RET applications as donor emitters [10,11]. UCNPs are capable of converting low energy near-infrared (NIR) light to higher energies (UV to NIR) via stepwise multiphoton adsorption and energy transfer processes in lanthanide ions, all happening prior to the RET [12].

The interest in RET applications involving UCNP has steadily increased over the past few years, but there remain many questions to be answered. In most studies the energy transfer process is partly based on RET, but often also contains contributions from reabsorption [13,14]. Some studies with in-depth investigation of UCNP in RET applications have been recently published, and the influence of various factors such as size [15], structure [16], and dye/UCNP ratio [17] have been discussed.

**Figure 1:**
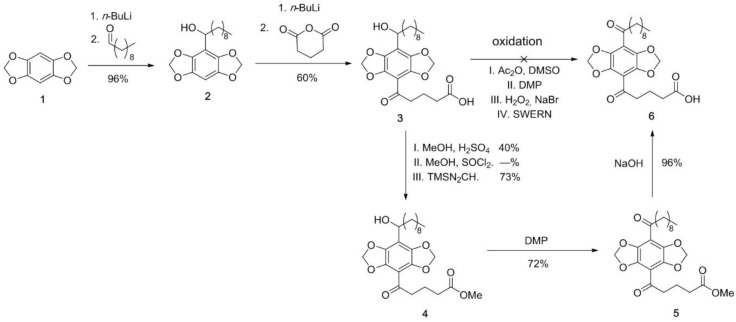
Synthesis of DBD-6 (DMP = Dess–Martin periodinane).

**Figure 2:**
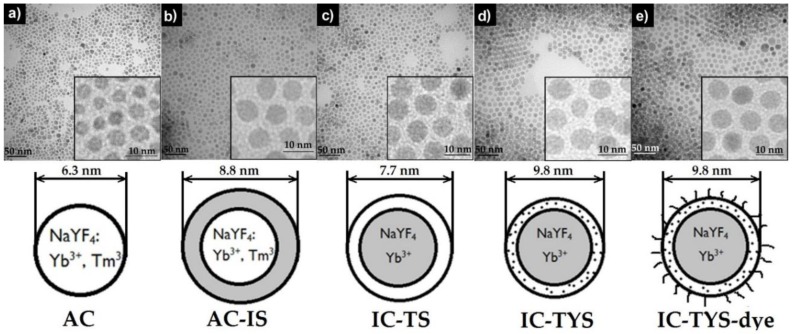
TEM images of NaYF_4_: Yb^3+^, Tm^3+^ UCNP: (**a**) active core (AC), (**b**) active core-inert shell (AC-IS), (**c**) inert core-Tm^3+^ shell (IC-TS), (**d**) inert core-Tm^3+^ and Yb^3+^ shell (IC-TYS), (**e**) inert core-Tm^3+^ and Yb^3+^ shell with dye (IC-TYS@dye). The scale bar is 50 nm (left) and 10 nm (right).

In this study, four types of NaYF_4_-based UCNP co-doped with Yb^3+^ and Tm^3+^ as sensitizer and activator, respectively, were investigated. The activator was either doped in the core or the shell of the nanoparticles: two species had Tm^3+^ in the core—simple active core-only (AC) UCNP and active core-inert shell (AC-IS)—which were also decorated with a passivating NaYF_4_ shell. Two other UCNP samples had inert cores doped only with Yb^3+^, while Tm^3+^ ions (inert core-Tm^3+^ shell, IC-TS) or both Yb^3+^ and Tm^3+^ (inert core-Tm^3+^ and Yb^3+^ shell, IC-TYS) were incorporated into the shell. The UCNP were used as donors for DBD-6 dye (Figure 1) attached to the surface of UCNP (Figure 2). DBD-6 belongs to the novel class of DBD dyes, which show unique photophysical properties such as very large Stokes shift, small overall molecular size, and high photochemical stability. As a toolbox, DBD dyes can be tailored for specific applications, like in the present case to substitute oleic acid as a capping ligand. The acceptor dyes were implemented via ligand exchange and had a tangential transition dipole moment relative to the UCNP surface. In order to have an internal reference, the UCNP were also co-doped with Er^3+^.

## 2. Materials and Methods

### 2.1. Materials

The rare earth chlorides RECl_3_ • 6 H_2_O (RE: Y^3+^; Yb^3+^; purities > 99.9%) and oleic acid (OA, 90% purity) were purchased from Alfa Aesar (Havelhill, MA, USA), Tm^3+^ chloride (TmCl_3_ • 6 H_2_O, >99.9%), ammonium fluoride (NH_4_F, 99%), cyclohexane (99.5%) and 3-(N-Morpholino)propanesulfonic acid (MOPS) were purchased from Sigma Aldrich (San Luis, AZ, USA). Ethanol (≥99.8%) and sodium hydroxide (NaOH, ≥99%) were purchased from Carl Roth (Karlsruhe, Germany), and Therminol^®^66 was purchased from FRAGOL GmbH+Co. KG (Mülheim, Germany). Sodium oleate was synthesized by the authors using NaOH, ethanol and OA. All chemical reagents were used as received without further purification.

For the synthesis of DBD-6, n-BuLi (1.6 M in hexane), decanal (95%), trimethylsilyldiazomethane (2 M in hexane), NaOH (98%), methanol (>99%) and tetrahydrofuran (>99.5%) were purchased from Acros organics (Morris, AL, USA), acetonitrile (>99.5%) and hydrochloric acid (HCl, 37%) from VWR (Randor, PA, USA), and glutaric acid anhydride was purchased from ABCR (Karlsruhe, Germany).

### 2.2. Synthesis of NaYF_4_ Based Core and Core-Shell UCNPs with Different Mole Ratios of Lanthanide(III) Ions

The UCNP synthesis was performed according to a protocol using Therminol^®^66, promising a significantly shortened synthesis time and is describe briefly in the following paragraphs [18]. The UCNP investigated had the Yb/Tm as the sensitizer/activator pair in common. In addition, Er^3+^ was added and served as an internal reference, because it is not influenced by the presence of the DBD-6, since no spectral overlap between the Er^3+^ emission and dye absorption is present.

#### 2.2.1. Synthesis of NaYF_4_ Core-Only UCNPs

All metal chlorides (0.8 mmol YCl_3_, 0.19 mmol YbCl_3_, 0.01 mmol TmCl_3_), 0.005 mmol ErCl_3_, 8 mL/37.8 mmol OA, and 12 mL Therminol^®^66 were transferred into a 100 mL three-necked flask and evacuated for 45 min at 140 °C. The reaction mixture was cooled down to 50 °C. 2.5 mmol sodium oleate and4.0 mmol ammonium fluoride were added under argon counter stream. The flask was evacuated for 30 min at 50 °C until all solids had dissolved. The reaction mixture was heated up to 320 °C (heat rate: 25 °C/min) and kept at 320 °C for 15 min. Then, the dispersion was cooled down to approximately 50 °C using a water bath. Purification was performed by adding 50 mL ethanol (at least of 99% purity or absolute) to the reaction mixture. The dispersion was divided equally into two centrifuge tubes (50 mL) and the particles were precipitated by centrifugation at 3100× *g* for 6–10 min. The precipitates were united and washed two additional times with ethanol (25 mL each time). The purified core-nanoparticle precipitates were re-dispersed in cyclohexane (15 mL to 20 mL).

#### 2.2.2. Preparation of the Shell Material

All necessary metal chlorides (YCl_3_, YbCl_3_, TmCl_3_; respective to the chosen shell type), OA (4 mL [12.6 mmol]) and 8 mL Therminol^®^66 were transferred into a 100 mL three-necked flask and heated to 140 °C for 45 min under vacuum. The reaction mixture was cooled down to 50 °C, 2.5 mmol sodium oleate and 4 mmol ammonium fluoride were added and evacuated while stirring at 50 °C for 30 min until no solids were left. The flask was then vented with Argon and the precursor material was stored in a refrigerator.

#### 2.2.3. Synthesis of Core-Shell-UCNPs

Sixty milligrams of core-UCNPs dispersed in cyclohexane, OA (25.2 mmol, 8 mL) and 12 mL Therminol^®^66 were transferred into a 100 mL three-necked flask. Cyclohexane was removed by evacuation and heating to 75 °C for 30 min. The reaction mixture was vented with argon, heated up to 305 °C (heat rate: 25 °C/min) and the shell precursor material was added via slow injection using a syringe pump (infusion rate: 2 mL/h, from Cole-Parmer^®^ 200 Touch Screen Series Syringe Pumps). As soon as the syringe was empty, the temperature was maintained at 305 °C for an additional 5 min. The reaction was stopped by cooling down to approximately 75 °C with a water bath. Purification was performed by adding 50 mL ethanol (at least of 99% purity or absolute). The particles were precipitated by centrifugation at 3100× *g* for 6–10 min. Purification was performed by washing two additional times with ethanol.

### 2.3. Synthesis of DBD-Labelled Fatty Acid (DBD-6)

The first functionalization of the DBD **1** was realized by lithiation followed by reaction with decanal in a very good yield, giving the alcohol **2**. The second functionalization was carried out by adding 2 eq. n-BuLi followed by glutaric anhydride, to afford acid 3 in 60%. Typical oxidation methods like Albright–Goldman, Dess–Martin–Periodinan (DMP) or Swern conditions did not lead to target compound **5**. As the synthesis of 6 using standard oxidation methods was not successful, we suspect that the acid has a disturbing influence on the reaction. To solve this problem we first protected the acid group by reaction with TMS-diazomethane in 73% yield, giving ester **6**. Subsequent oxidation with DMP afforded 5 with a yield of 72%. Finally, the deprotection of 5 with NaOH to acid **5** proceeded with a 96% yield. For further information please refer to Appendix A. 

### 2.4. Ligand Exchange with DBD-6

For the ligand exchange reaction, 2.5 mg of the dye was dispersed in 10 mL cyclohexane. Respective volumes of UCNP solutions in cyclohexane were added and the mixture was incubated for 24 h under vigorous stirring at 600 rpm.

### 2.5. Steady-State and Time-Resolved Upconversion Luminescence Measurements at Room Temperature

Steady state and time resolved upconversion luminescence measurements were carried out at room temperature. The UCNP were excited at 976 nm using a wavelength tunable pulsed Nd:YAG laser (Quanta Ray, Spectra-Physics, Mountain View, CA, USA) combined with an OPO system (GWU-Lasertechnik Vertriebsges.mbH, Erftstadt, Germany) operating at 10 Hz as the excitation light source (26 mJ/130 mW). All upconversion luminescence spectra were recorded using an intensified CCD-camera (iStar DH720-18V-73, Andor Technology, Belfast, Great Britain) coupled to a spectrograph (Shamrock SR 303i, Andor Tehcnology, Belfast, Great Britain) equipped with a 600 L/mm grating. The time resolved luminescence spectra was recorded with applying “boxcar” technique in which the amplitude $A_{i}$ is converted to “true amplitude” $F_{i}$ because of the dependence of $A_{i}$ on the detection gate width $t_{gate}$ [19]. Equation (1) shows this relation:

(1) $$F_{i}=\frac{A_{i}}{\tau_{i}\left(1-e^{-\frac{t_{gate}}{\tau_{i}}}\right)}$$

where $F_{i}$ is the coefficient by gate width $t_{gate}$ correction, $A_{i}$ is the experimental coefficient (amplitude of the fit function), $t_{gate}$ is the detection gate width (duration of the photoluminescence emission recording), and $\tau_{i}$ is the luminescence decay time of the *i* component. For fitting of recorded luminescence decay curves, Equation (2) was used:

(2) $$I\left(t\right)=y_{0}+A_{1}e^{-k_{1}t}+A_{2}e^{-k_{2}t}$$

where $k_{i}=\frac{1}{\tau_{i}}\mathrm{is}\;\mathrm{the}\;\mathrm{rate}\;\mathrm{constant}$, I(t) is the luminescence intensity in dependence on time t, $y_{0}$ is the offset of the fitting function and accounts for background signal contribution, and $\tau_{i}$ is the luminescence decay time of the *i* component.

The relative fractions $f_{i}$ were calculated using the following equation:

(3) $$f_{i}=\frac{F_{i}\;\tau_{i}}{\sum_{k}F_{k}\tau_{k}}.$$

### 2.6. UV/Vis Absorption Measurements

The UV/Vis absorption measurements of DBD-6 dye in cyclohexane were performed in a 1-cm quartz cuvette with a volume of 3 mL using a Lambda 750 UV/VIS Spectrometer from PerkinElmer (Shelton, CT, USA). The absorption was scanned from 300 to 550 nm using a slit width and step width of 1 nm and recorded by operating UV WinLab Software (version 5.2.0). The baseline was corrected by measuring absorption of neat cyclohexane.

### 2.7. Calculation of the Number of DBD-6 Molecules per Single UCNP

The DBD-6 dye was dissolved in cyclohexane (0.02 mg/mL). To each 1 mL sample of the dye, 1.5 mL of UCNP with decreasing concentration (0.92, 0.46, 0.23, 0.092, and 0 mg/mL) were added. The mixtures were stirred overnight and centrifuged at 1500× *g* for 3 min. In the supernatant, the absorption of the dye was measured and used to determine the amount of DBD-6 bound to the UCNP.

The following formula was used to estimate the number of dye molecules per UCNP:

(4) $$n=\frac{\frac{\left(c_{0}-c_{d}\right)*v}{M_{d}}*N_{a}}{\eta \left(UCNP\right)},$$

where *c*_0_ is the starting concentration of the dye, *c_d_* is the concentration of the dye in the supernatant, *v* is sample volume, *M_d_* is the molar mass of the dye (434 g/mol), *N_a_* is Avogadro constant (6.02 × 10^23^), and *η*(*UCNP*) is the number of UCNP added to the sample, which was calculated according to [20]. Assuming that our UCNP were roughly spherical in shape (see Figure 2), we used the following formula to calculate the mass of one UCNP:

(5) $$m=\rho \pi r^{3},$$

where *ρ* (g/cm^3^) is the density of UCNP [20], and *r* is the average radius of one UCNP (see Table 2). The number of UCNP in one sample was calculated as the ratio between the mass of UCNP added to the sample to the mass of one UCNP.

Correction using control samples without the dye and baseline correction with pure cyclohexane were performed. UCNP concentration was held equal and constant for spectral measurements of all samples.

### 2.8. Resonance Energy Transfer (RET)

The Förster distance range of nonradiative energy transfer was estimated using Equation (6) [4,21]:

(6) $$R_{0}=0.02108{\left(\kappa^{2}\;\Phi n^{-4}J\right)}^{1/6}\;\mathrm{nm}$$

where $\kappa^{2}$ = 0.67 (for dynamic averaging, which could be questioned in case of the donor being a part of a capping ligand) is the dipole orientation factor, *Φ* is the quantum yield of the donor, *n* = 1.42 is the refractive index of cyclohexane, and *J* (M^−1^ cm^−1^ nm^4^) represents the spectral overlap between the area-normalized donor (Tm^3+^) photoluminescence and acceptor (DBD-6) absorption spectra (see Equation (7)):

(7) $$J=\int_{430}^{500}F\left(Tm^{3+}\right)\varepsilon_{DBD-6}\lambda^{4}d\lambda .$$

### 2.9. Structural and Size Investigations

The UCNP were investigated using a transmission electron microscope (TEM, Tecnai G2 20, from FEI/Thermo Fisher Scientific (Hillsboro, OR, USA)) and an acceleration voltage of 200 kV. Particle counting was performed using ImageSP Viewer software. 

X-ray powder diffraction (XRD) patterns were collected using a D5005 instrument (Siemens AG, Munich, Germany) in a range of 3–70°/2θ with divergence aperture, scattering ray aperture and graphite monochromatized CuK_α_ radiation (*λ* = 0.15406 nm). The scanning step was 0.02°/2θ with a counting time of 4 s per step.

## 3. Results

### 3.1. Synthesis and Characterisation of the DBD-6 Dye

The first functionalization of the DBD **1** was realized by lithiation and a further reaction with octanal in a very good yield. The second functionalization was carried out by adding 2 eq. n-BuLi and glutaric anhydride to afford the acid **3** in 60%. Typical oxidation-methods like Albright-Goldman, Dess-Martin-Periodinan (DMP) or Swern-conditions did not lead to product **6**.

As the synthesis of **6** due to the standard-oxidation methods was not successful, we suspect that the acid has a disturbing influence on the reaction. Solving that problem we protected the acid group by Fischer-esterification in 40% to **4**. The synthesis could not be optimized by building the acid chloride. By using TMS-diazomethane, product **4** was isolated in a good yield of 73%. Oxidation with DMP afforded **6** in 72%. Finally the deprotection of **5** with NaOH to acid **6** proceeded in a 96% yield.

Photophysical properties of DBD-6 are summarized in Table 1. 

### 3.2. Design and Synthesis of Dye-Labeled UCNP

The first type of UCNP consisted of active core-only (AC) NaYF_4_: Yb^3+^, Tm^3+^ nanoparticles (plus traces of the internal reference Er^3+^—since this applies for all other UCNP it will not be stated explicitly from now on). This kind of nanoparticle is known to provide low upconversion luminescence intensity due to surface quenching, so a protective shell is often used to improve the emission intensity [22]. The tight control of the experimental conditions allowed to synthesize tailored shells of only 1–2 nm thickness (see Table 2 as well as Figure 2 for particle size information). For the second sample, we used an inert (undoped) NaYF_4_ shell with a thickness of about 2 nm to test the influence of surface quenching as well as the increase of the donor–acceptor distance on the energy transfer (ET) process. These UCNP will be referred to as active core-inert shell (AC-IS).

Since in active core particles Tm^3+^ ions are present on the surface as well as in the bulk of a nanoparticle, individual donor–acceptor distances are dependent on the respective location of Tm^3+^ relative to the surface, and a distribution of distance has to be taken into account. To limit the distance between the pairs, we designed two additional kinds of UCNP with no Tm^3+^ ions in the core and only Tm^3+^ or Tm^3+^ and Yb^3+^ ions in a sub-3 nm shell (see Table 2, Figure 2). The core of both species consisted of NaYF_4_: Yb^3+^ in both cases, and we will refer to these particles as inert core-Tm^3+^ shell (IC-TS) and inert core-Tm^3+^ and Yb^3+^ shell (IC-TYS). The presence of Yb^3+^ in the shell of only one of the species was to evaluate the influence of the distance between Tm^3+^ and Yb^3+^ ions on the efficiency of upconversion and the subsequent ET process. In the case of the IC-TS UCNP, the surface quenching of the sensitizer Yb^3+^ was eliminated as well.

The as-synthesized UCNP are normally capped with oleic acid (OA) and soluble in cyclohexane. The DBD-6 dye that we used as the RET acceptor roughly shares the same solubility characteristics as oleic acid—a hydrophilic carboxylic group and a hydrophobic hydrocarbon chain (see Figure 1, compound **6**). Therefore, we incubated the OA-capped UCNP with the dye solution and replaced OA molecules by DBD-6 molecules via ligand exchange (vide infra).

**Figure A1:**
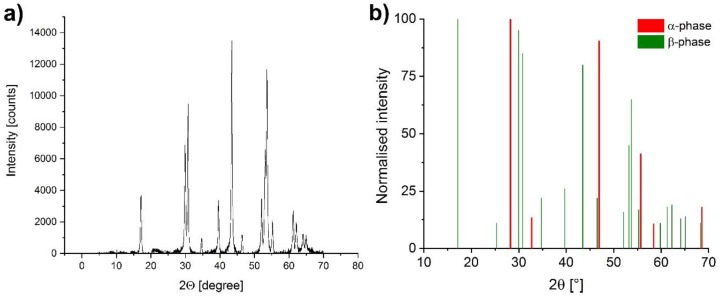
XRD diffraction patterns of AC UCNP indicating hexagonal crystal structure (**a**). Reference XRD reflection data origin from JCPDs No. 01-077-2042 (α-NaYF_4_) and JCPDs No. 00-028-1192 (β-NaYF_4_) (**b**).

As can be seen from Figure 2, the UCNP of the different sets are uniform in size and share a hexagonal lattice, as indicated by the TEM, XRD (Figure A1), and the spectral intensity distribution of the Er^3+^ luminescence in the green spectral region (vide infra) [23]. The addition of the dye did not influence the morphology or result in clustering of UCNP as can be seen from the TEM images (Figure 2d,e). Average UCNP sizes and their distributions of are presented in Table 2.

The approximate calculation of the number of dye molecules per UCNP revealed the following trend—with the increase of the UCNP concentration, the dye/UCNP number decreased almost tenfold (from 837 ± 93 to 92 ± 22). Based on these numbers, measurements of the dye absorption are an easily accessible parameter to control UCNP concentration in a sample.

We used Equation (6) to calculate the minimum and maximum Förster distance values *R*_0min_ and *R*_0max_ for the UCNP-DBD-6 RET pair, taking the overall upconversion luminescence quantum yield as *Φ* (estimated as *Φ*_min_ = 0.001 and *Φ*_max_ =0.01 according to reported literature values), resulting in *R*_0min_ = 2.1 nm and *R*_0max_ = 3.1 nm. This corresponds well with the values reported in literature for other UCNP RET pairs [21,24,25].

### 3.3. Luminescence Emission Spectra

**Figure 3:**
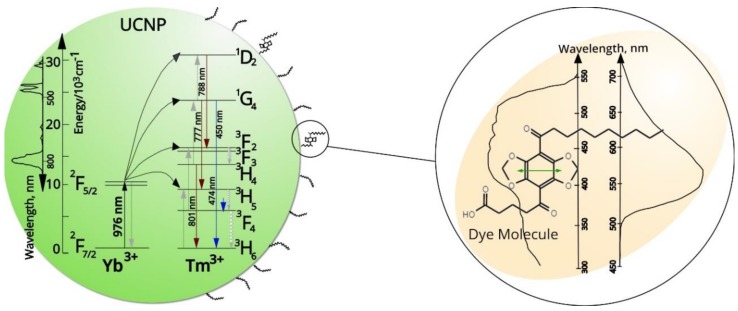
Schematic representation of the ligand exchange and energy transfer process between the DBD-6 dye and oleic acid-capped UCNP (left circle). Dye absorption (**left**) and emission (**right**) spectra and (right circle) UCNP emission overview are shown in black on the right side of the image. Transition moments of the DBD are shown as green arrows. Tm^3+^ emission spectra and energy level diagrams are shown on the left. The thickness of OA layer is ~1 nm and the distance between the UCNP surface and the DBD dye is ~0.5 ± 0.2 nm.

When NaYF_4_: Yb^3+^, Tm^3+^ UCNP are excited at 976 nm, ^2^F_7/2_→^2^F_5/2_ transition of trivalent ytterbium results into several Tm^3+^ emission peaks—at 451 nm (assigned to ^1^D_2_→^3^F_4_ transition), at 475 nm (^1^G_4_→^3^H_6_), and at 800 nm (^3^H_4_→^3^H_6_) [26]. The absorption peak maximum of the DBD-6 is in the range of 430–470 nm, which overlaps with the blue emission peaks for the ^1^D_2_→^3^F_4_ and ^1^G_4_→^3^H_6_ transitions from UCNP at 451 and 475 nm (see Figure 3), respectively. Furthermore, the emission peak of the DBD-6 dye is quite broad and its maximum can be found around 550 nm (see Figure 3).

**Figure 4:**
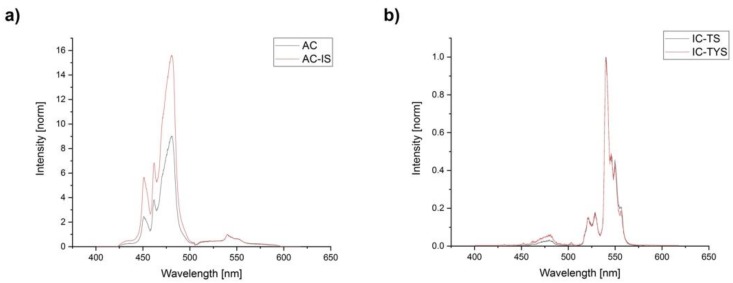
Upconversion photoluminescence spectra (intensity-normalized at 550 nm of AC and AC-IS (**a**), IC-TS and IC-TYS (**b**) upon 976 nm excitation.

The upconversion luminescence spectra of all UCNP species with 976 nm excitation were normalized to the erbium reference luminescence band around 550 nm (see Figure 4). The strongest blue luminescence (^1^D_2_→^3^F_4_ and ^1^G_4_→^3^H_6_ transitions) was observed for the AC-IS species, which corresponds to data reported in literature—a passivating shell protects Tm^3+^ ions from quenching by the solvent [22]. It was followed by AC species, where Tm^3+^ ions were equally distributed in/on the UCNP—ions at the surface were quenched while those in the bulk were not. IC-TS and IC-TYS have shown almost equally weak blue luminescence, although the species with Yb^3+^ in the shell slightly surpassed the IC-TS UCNP. This indicates two points: first, in samples with Tm^3+^ ions only in the outer layer surface quenching is especially strong for bands with 3- and 4-photon transitions, hence the much stronger Er^3+^ band at 550 nm. Second, the distance between sensitizer and activator ions plays a role in the upconversion process, since the particles with Yb^3+^ ions not only in the core, but also in the shell (IC-TYS) have shown a slight increase in the blue band luminescence intensity compared to IC-TS. The elimination of the surface-related Yb^3+^-quenching does not compensate the decrease in the ETU due to larger distances between activator and sensitizer, especially in case of three- or four-photon-induced processes.

**Figure 5:**
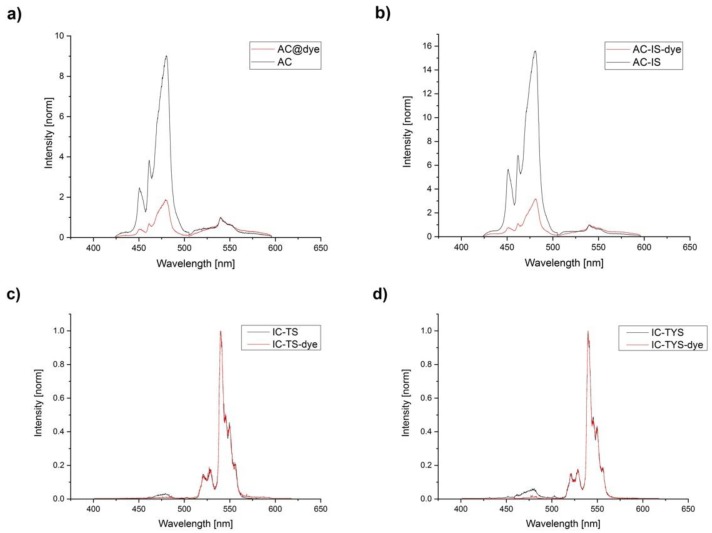
Upconversion photoluminescence spectra (intensity-normalized at 550 nm of AC (**a**), AC-IS (**b**), IC-TS (**c**) and IC-TYS (**d**) upon 976 nm excitation. For AC and AC-IS species, the number of DBD-6 molecules per UCNP amounted to 92 ± 22, for IC-TS and IC-TYS-138 ± 39.

A prominent intensity drop of blue emission band was seen for all dye-labelled samples (see Figure 5, the chosen dye concentrations were motivated by the theoretical prevalence of RET mechanisms over readsorption on one hand and on the solubility of the dye in cyclohexane on the other). The blue/reference band ratios dropped up to 83% (Table 3). Such drops in blue band emission can be attributed to resonance energy transfer (RET), since inner filter effects would account for a much smaller drop (no more that 5%) [17]. AC-IS species showed the strongest luminescence at 470 nm, but also the biggest drop after the addition of the dye. They were closely followed by the AC species, which didn’t have a protective shell. The “inert core family” showed the smallest drops in blue/reference band ratios, although the blue luminescence was extremely weak to begin with and the number of dye molecules per UCNP was larger (see Figure 5). Due to the close proximity of all Tm^3+^ ions to the surface in these samples, they were most prone to surface quenching, which might have competed with energy transfer to the dye. 

As can be seen from the spectra (Figure 5a,b), the peak assigned to ^1^D_2_→^3^F_4_ transition was more affected by introduction of the dye, due to the better spectral overlap with DBD-6. Besides, in samples labelled with DBD-6 dye, a wide peak (around the reference peak) appeared, which was attributed to the dye emission. In reference experiments with samples containing only DBD-6, no dye emission was found for 976 nm excitation.

### 3.4. Luminescence Decay Kinetics of the Dye-Labelled UCNP

**Figure 6:**
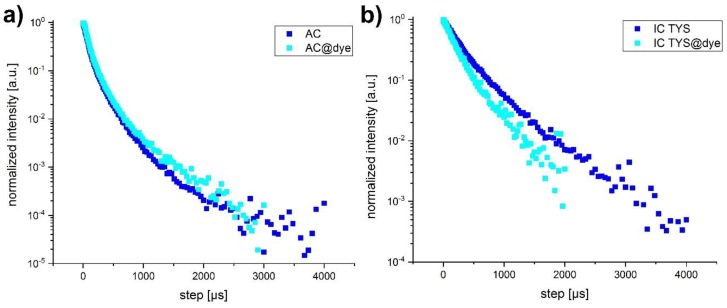
Upconversion luminescence decay of the blue emission band (^1^D_2_→^3^F_4_ and ^1^G_4_→^3^H_6_ transition) of quenched (cyan) and non-quenched (blue) AC (**a**) and IC TYS (**b**) UCNP upon 976 nm excitation.

Luminescence decay kinetics of the different UCNP samples were measured upon 976 nm excitation for the blue (451 and 475 nm) and the reference (550 nm) emission bands (see Figure 6 for examples of luminescence decay kinetics). Luminescence lifetime values were determined by fitting the decays using a biexponential model (see Equation (2)). The shorter decay component (*τ*_1_) is usually assigned to Tm^3+^ atoms located closer to the nanoparticle surface and thus more susceptible to surface quenching by the solvent. The longer decay component (*τ*_2_) is attributed to the “bulk” Tm^3+^ atoms. Both values were consistent with those found in literature (tens to hundreds of µs) [27].

From Table 4 it can be seen that the short components of photoluminescence decay kinetics of the blue emission band were faster in dye-labelled samples (shorter decay times). For the “reference” band no significant effect was observed (AC and IC-TS were not affected by the presence of DBD-6 dye, while AC-IS and IC-TYS showed a slight decrease, that can be neglected, given the margin of error). Since the long decay components are normally attributed to the luminescence of the “bulk” Tm^3+^ ions, which had a larger distance to the DBD-6 (RET acceptor), we considered their contribution to RET rather small and consequently used only the surface related luminescence fraction for the further calculations.

We calculated the apparent RET efficiency of the UCNP–dye system, using data from non-labeled UCNP as a reference, via the classic equation for single donor–single acceptor pairs: [16,17]

(8) $$\eta =1-\frac{\tau_{with\;dye}}{\tau_{no\;dye}}.$$

**Figure 7:**
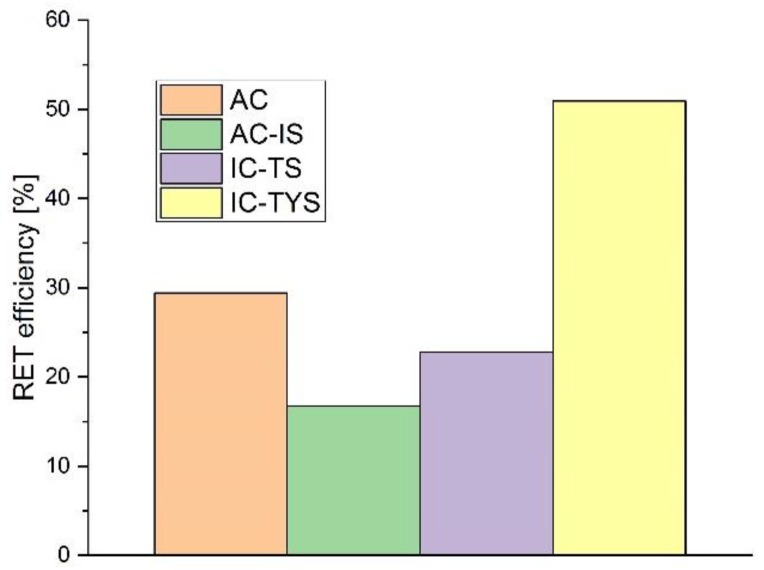
RET efficiencies calculated from the short components of decay lifetimes of blue emission band of the UCNP-dye system. Equation (8) was used to calculate the efficiencies.

Short components of the blue band were used to calculate the RET efficiency (Figure 7). The highest values were obtained for the sample with an inert core and both Tm^3+^ and Yb^3+^ ions in the shell (IC-TYS), despite the low overall brightness of UCNP with such an architecture. This was somewhat expected, since this UCNP species has all donor ions close to the surface, and a shorter distance between sensitizer and activator ions. Addition of an inert shell seemed to decrease the RET efficiency in the sample with an active core (despite an increase of upconversion efficiency), which could be explained by an increase in RET donor–acceptor distance. 

**Figure A2:**
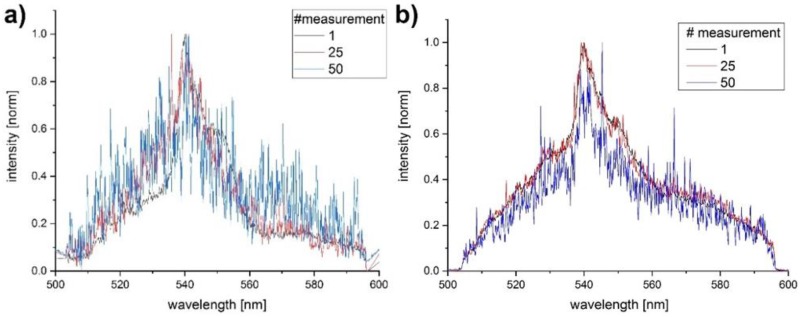
Reference/dye emission peak evolution with time of (**a**) AC, (**b**) AC-dye UCNP.

We also compared the evolution of peak shapes of quenched and non-quenched UCNP. As can be seen from Figure 5 and Figure A2, the shape of 550 nm emission bands changed after the dye was introduced to the nanoparticles. A wider peak/shoulder appeared around the “reference” band (550 nm), most apparently in AC and AC-IS, as the ratio of the reference to blue emission band was lower. The lifetime of the DBD-6 dye hardly reached 20 ns (when directly excited and measured with time-correlated single photon counting, see Table 2), while UCNP luminescence lifetimes reached up to hundreds of µs. Thus, the wide emission band—which we attribute to the presence of the dye—would disappear within nanoseconds if the RET process was not in place. But the difference in reference peak shape of UCNP and UCNP-dye is still obvious even after tens of µs, which also serves as direct evidence for the active RET. 

## 4. Discussion

In this study, we investigated several samples of core-shell and core-only UCNP for their photophysical properties with respect to energy transfer to DBD-6, which belongs to a novel class of DBD dyes. DBD dyes are relatively small molecules with unique photophysical properties such as very large Stokes shifts (see Table 2) and outstanding photostability. The functionality of the dyes can be adapted to specific needs, allowing further functionalization, e.g., for biosensing [28,29].

TEM measurements of UCNP indicated high homogeneity in their size and shape, ranging from 6.3 ± 1.4 nm for core UCNP to 9.8 ± 1.5 nm for the core-shell species, with a shell thickness of ~2.5 nm.

Basic investigations of the upconversion behavior of the UCNP species revealed AC-IS particles to be the brightest due to the inert shell protecting Tm^3+^ ions from quenching by environment (a comparison was made with identical measurement conditions with respect to laser excitation and UCNP concentration). UCNP with Tm^3+^ ions only in the shell (IC-TS and IC-TYS) showed the lowest overall brightness, but their luminescence lifetimes were surprisingly high. Similar phenomena were described in [16].

The possibility of RET between UCNP and the DBD-6 attached to their surface was discussed, suggested by significant decreases in the blue/reference band ratios in samples labelled with the dye. RET efficiencies were calculated from luminescence decay data, and the efficiency of IC-TYS nanoparticles was revealed to be the highest despite the low brightness of the sample. The addition of an inert shell decreased the efficiency of the active core UCNP by almost half. In the present case, the DBD-6 was designed to substitute OA with minimal effect on the capping agent structure. It is therefore attractive to assume that DBD-6 is oriented parallel to the OA molecules. As a result, the transition dipole moment of the dye (see Figure 3) would be placed tangentially relative to the UCNP surface. This might explain why the experimentally found RET-efficiencies always tend to be smaller than the ones that could be calculated based on the photophysical parameters: e.g., for AC the RET-efficiency was expected to be 73% (averaging over all Tm^3+^ ions). But of course, in the calculation a dipole–dipole interaction was assumed, which is probably not the best approximation for lanthanide ions (on the other hand, the multipole character might help to be less dependent on *κ*^2^). Therefore, theoretical calculations are limited by the fact that a number of parameters, such as quantum yields, are not fully known.

## Figures and Tables

**Table 1:** Photophysical properties of the DBD-6 dye.

Solvent	*λ*_Abs_ [nm]	*λ*_Em_ [nm]	Δ*λ* [nm]	*τ_F_* [ns]	*ε* [M^−1^cm^−1^]	*φ_F_*
DCM	435	547	112	26.1	2970	0.66
ACN	422	560	138	21.9	2890	0.51
methanol	436	605	169	4.6	2550	0.08
H_2_O	463	557	94	8.1 ^a^	2530	0.09

^a^ <*τ_F_*>, 4.9 ns (35.0%), 9.0 ns (65.0%).

**Table 2:** Diameters (as determined by TEM) of various UCNP species in cyclohexane.

Sample	AC	AC-IS	IC-TS	IC-TYS	IC-TYS@dye
Diameter, nm	6.3 ± 1.4	8.8 ± 1.7	7.7 ± 1.6	9.8 ± 1.5	9.8 ± 1.5

**Table 3:** Blue/reference band intensity ratios for all UCNP species with and without DBD-6.

UCNP Species	No Dye	With Dye
AC	5.8	1.1
AC-IS	10.1	1.7
IC-TS	0.15	0.11
IC-TYS	0.13	0.04

**Table 4:** Luminescence decay times with respective fractions (in parentheses) of AC, AC-IS, IC-TS and IC-TYS UCNP (blue = ^1^D_2_→^3^F_4_ and ^1^G_4_→^3^H_6_ transition) upon 976 nm excitation (as calculated from PL decay curves, e.g., in Figure 6). *τ*_1_ is the short component of the luminescence decay time, *τ*_2_ is the long component and *τ*_av_ is the average time of luminescence decay.

*τ*, µs/Fraction, %		AC	AC @dye	AC-IS	AC-IS @dye	IC-TS	IC-TS @dye	IC-TYS	IC-TYS @dye
blue (470 nm)	*τ*_1_	119 (77%)	84 (77%)	173 (78%)	144 (92%)	228 (65%)	176 (69%)	161 (45%)	79 (65%)
	*τ*_2_	308 (23%)	263 (23%)	387 (22%)	483 (8%)	567 (35%)	590 (31%)	582 (55%)	259 (35%)
	***τ*_av_**	**163 ± 26**	**125 ± 9**	**221 ± 30**	**171 ± 33**	**348 ± 71**	**305 ± 93**	**391 ± 44**	**141 ± 30**
reference (550 nm)	*τ*_1_	68 (82%)	69 (82%)	132 (84%)	116 (93%)	64 (86%)	61 (78%)	92 (58%)	57 (48%)
	*τ*_2_	233 (18%)	225 (18%)	328 (16%)	369 (7%)	214 (14%)	169 (22%)	479 (42%)	338 (52%)
	***τ*_av_**	**98 ± 8**	**97 ± 8**	**164 ± 23**	**135 ± 24**	**85 ± 4**	**85 ± 5**	**254 ± 12**	**204 ± 8**

## Appendix A

### Appendix A.2. DBD-6 Synthesis Intermediates

All synthesis were carried out in a nitrogen atmosphere with standard Schlenk techniques. Reagents and solvents were purchased from commercial sources as listed below and used without further purification. The NMR spectra of the compounds were recorded at a Bruker Avance 300 MHz in deuterated solvents as internal standard (^1^H NMR: CDCl_3_ = 7.26 ppm, ^13^C NMR: CDCl_3_ = 77.2 ppm, [D6] DMSO = 39.5 ppm). All chemical shifts are given in ppm. Couplings constants (J) are given in Hertz (Hz). Fine structure analysis was performed and multiplicities were abbreviated as: s = singlet, d = doublet, t = triplet, m = multiplet. IR spectra were recorded on a Perkin Elmer FTIR spectrometer Spectrum 2. The melting points have been determined at an Elektrothermal 9100 melting point apparatus and are uncorrected. High resolution mass spectrometry (HRMS) was performed with impact ionization (EI) equipped with a quadrupole. Flash chromatography was performed using silica gel 60 (50–63 mm). All reactions were monitored by thin layer chromatography (TLC). Compound **1** was synthesized according to [30], Dess–Martin–Periodinan (DMP) was synthesized according to [31].

1-(Benzo[1,2-d:4,5-d′]bis([1,3]dioxole)-4-yl)decan-1-ol (**2**)

Benzo[1,2-d:4,5-d′]bis[1,3]diioxole (DBD) **1** (405 mg, 2.44 mmol, 1.0 eq.) was dissolved in 30 mL dry THF, cooled to −40 °C and *n*-BuLi (1.6 M in hexane, 2.30 mL, 3.66 mmol, 1.5 eq.) was added. After stirring at −40 °C for 2 h decanal (0.55 mL, 2.93 mmol, 1.2 eq.) was added to the yellow solution and stirred for another 2 h. The reaction mixture was warmed to room temperature and quenched with 1 M HCl. After phase separation the water-layer was extracted with DCM (3x). The combined organic layers were dried over MgSO_4_, evaporated and the resulting residue was purified by flash silica gel column chromatography (PE:EE 5:1) to yield **2** (753 mg, 2.34 mmol, 96%) as a white solid, m.p. 58.8–59.5 °C, ^1^H NMR (300 MHz, CDCl_3_): δ = 6.41 (s, 1H), 5.58 (s, 4H), 4.81 (t, ^3^*J* = 7.4 Hz, 1H), 1.95–1.71 (m, 2H), 1.25 (s, 14H), 0.87 (t, ^2^*J* = 9.1 Hz,3H) ppm. ^13^C NMR (75 MHz, CDCl_3_): δ = 141.4, 138.7, 111.7, 101.4, 92.2, 67.9, 37.2, 32.0, 29.7, 29.5, 29.4, 25.8, 22.8, 14.3 ppm. IR (ATR) $\tilde{v}$ = 3407, 3323, 2915, 2850, 1453, 1322, 1165, 1121, 1054, 1030, 923 cm^−1^. HRMS: calcd. for C_18_H_26_O_5_ 322.1780 [M]^+^; found 322.1767.

5-(8-(1-Hydroxydecyl)benzo[1,2-d:4,5-d′]bis([1,3]dioxole)-4-yl)-5-oxopentanoic acid (**3**)

Compound **2** (250 mg, 0.78 mmol, 1.00 eq.) was solved in 20 mL dry THF and cooled to −40 °C. *n*-BuLi (1.6 M in hexane, 1.02 mL, 1.63 mmol, 2.1 eq.) was added dropwise to the reaction mixture and stirred for 1 h at −40 °C. After adding glutaric acid anhydride (106 mg, 0.93 mmol, 1.2 eq.) the solution turned green and was stirred for another hour while the solution reached room temperature. The mixture was quenched with H_2_O. The water layer was extracted several times with DCM. After drying over MgSO_4_ all solvents were removed in vacuo. The resulting residue was purified by flash silica gel column chromatography (DCM:MeOH 25:1) to yield **3** (200 mg, 0.46 mmol, 60%) as a yellow oil. ^1^H NMR (300 MHz, CDCl_3_): δ = 6.42 (s, 1H), 5.58 (s, 4H), 4.08 (t, ^3^*J* = 6.7 Hz, 1H), 2.60–2.22 (m, 4H), 2.13–1.75 (m, 4H), 1.27 (s, 14H), 0.87 (s, 3H) ppm. ^13^C NMR (75 MHz, CDCl_3_): δ = 178.5, 172.3, 141.4, 139.2, 107.8, 101.5, 92.8, 69.7, 33.4, 32.0, 29.6, 29.4, 29.3, 25.6, 22.8, 19.9, 14.2 ppm. IR (ATR) $\tilde{v}$ = 3419, 2922, 2853, 1709, 1679, 1655, 1435, 1305, 1071, 937 cm^−1^. HRMS: calcd. for C_23_H_32_O_8_ 436.2097 [M]^+^; found 436.2081.

Methyl 5-(8-(1-hydroxydecyl)benzo[1,2-d:4,5-d′]bis([1,3]dioxole)-4-yl)-5-oxopentanoate (**5**)

To a solution of 8 mL benzene and 2 mL methanol was 3 (220 mg, 0.50 mmol, 1.00 eq.) added at 0 °C. After adding Trimethylsilyldiazomethane (2 M in diethylether, 0.5 mL, 0.50 mmol, 2.0 eq.) the solution was stirred for 20 min at 0 °C. The solvents were evaporated and the crude mixture diluted with 20 mL 1 M CH_3_COOH and 20 ML DCM. After phase separation the water layer was extracted two times with DCM. The combined organic layers were washed with Brine, dried over MgSO_4_ and all solvents removed in vacuo. Purification of the residue by flash silica gel column chromatography (DCM:MeOH 25:1) afforded **5** (195 mg, 0.37 mmol, 73%) as a red oil. ^1^H NMR (300 MHz, CDCl_3_): δ = 5.98 (s, 4H), 4.79 (t, ^3^*J* = 7.0 Hz, 1H), 3.65 (s, 3H), 2.95 (t, ^3^*J* = 7.0 Hz, 2H), 2.40 (t, ^3^*J* = 7.3 Hz, 2H), 2.09–1.92 (m, 2H), 1.88–1.68 (m, 2H), 1.23 (s, 14H), 0.85 (t, ^3^*J* = 3.2 Hz, 3H) ppm. ^13^C NMR (75 MHz, CDCl_3_): δ = 195.6, 173.8, 140.8, 139.3, 115.8, 106.9, 102.2, 67.8, 51.6, 42.5, 36.9, 33.2, 31.9, 29.6, 29.4, 29.3, 25.6, 22.8, 19.0, 14.2 ppm. IR (ATR) $\tilde{v}$ = 2925, 2855, 1736, 1433, 1303, 1200, 1171, 1067, 914 cm^−1^. HRMS: calcd. for C_24_H_34_O_8_ 450.2254 [M]^+^; found 450.2256.

Methyl 5-(8-decanoylbenzo[1,2-d:4,5-d′]bis([1,3]dioxole)-4-yl)-5-oxopentanoate (**6**)

DMP (268 mg, 0.63 mmol, 1.5 eq.) was added to a solution of **5** (190 mg, 0.42 mmol, 1.00 eq.) in 20 mL anhydrous DCM. The mixture was stirred overnight and washed three-times with a solution of NaHCO_3_/Na_2_S_2_O_3_ (250 g/L). The water layer was extracted several times with DCM. The combined organic layers were washed with Brine, dried over MgSO_4_ and concentrated in vacuo. The resulting residue was purified by flash silica gel column chromatography (PE:EE 2:1) to yield **6** (135 mg, 0.30 mmol, 72%) as an orange solid, m.p. 99.0–100.5 °C. ^1^H NMR (300 MHz, CDCl_3_): δ = 6.09 (s, 4H), 3.66 (s, 3H), 3.00 (t, ^3^*J* = 7.0 Hz, 2H), 2.90 (t, ^3^*J* = 7.3 Hz, 2H), 2.41 (t, ^3^*J* = 7.3 Hz, 2H), 2.11–1.89 (m, 2H), 1.79–1.55 (m, 2H), 1.25 (s, 12H), 0.86 (t, ^3^*J* = 5.9 Hz, 3H) ppm. ^13^C NMR (75 MHz, CDCl_3_): δ = 195.6, 173.7, 141.3, 141.2, 121.0, 110.4, 109.9, 102.7, 51.7, 43.9, 42.8, 33.1, 32.0, 29.6, 29.4, 29.3, 23.8, 22.8, 18.9, 14.2 ppm. IR (ATR) $\tilde{v}$ = 2922, 2852, 1738, 1673, 1473, 1431, 1283, 1076, 1027, 938 cm^−1^. HRMS: calcd. for C_24_H_32_O_8_ 448.2097 [M]^+^; found 448.2084.

5-(8-decanoylbenzo[1,2-d:4,5-d′]bis([1,3]dioxole)-4-yl)-5-oxopentanoic acid (**4**)

A solution of **6** (100 mg, 0.22 mmol, 1.00 eq.) in 4 mL acetonitrile and 4 mL 2M NaOH was heated for 2 h at 60 °C. The mixture was acidified with 1 M HCl to pH = 1 and extracted three-times with DCM. The combined organic layers were dried over MgSO_4_, evaporated and was purified by flash silica gel column chromatography (PE:EE 2:1) to yield **4** (95 mg, 0.22 mmol, 98%) as an orange solid, m.p. 168.5–169.5 °C, ^1^H NMR (300 MHz, CDCl_3_): δ = 12.06 (s, 1H), 6.14 (s, 4H), 2.95 (t, ^3^*J* = 7.1 Hz, 2H), 2.89 (t, ^3^*J* = 7.2 Hz, 2H), 2.28 (t, ^3^*J* = 7.3 Hz, 2H), 1.87–1.72 (m, 2H), 1.57 (s, 2H), 1.26 (12H), 0.87 (t, ^3^*J* = 6.3 Hz, 3H) ppm. ^13^C NMR (75 MHz, CDCl_3_): δ = 195.4, 174.1, 140.5, 140.4, 109.6, 109.3, 102.6, 42.9, 42.2, 32.6, 31.3, 28.9, 28.7, 28.5, 23.2, 22.1, 18.6, 14.0 ppm. IR (ATR) $\tilde{v}$ = 3021, 2921, 2853, 1727, 1668, 1474, 1434, 1282, 1077, 1030, 937 cm^–1^. HRMS: calcd. for C_23_H_30_O_8_ 434.1941 [M]^+^; found 434.1937.
